# GIS as an Epidemiological Tool to Monitor the Spatial–Temporal Distribution of Tuberculosis in Large Game in a High-Risk Area in Portugal

**DOI:** 10.3390/ani11082374

**Published:** 2021-08-11

**Authors:** José Aranha, Ana Carolina Abrantes, Raquel Gonçalves, Rui Miranda, João Serejo, Madalena Vieira-Pinto

**Affiliations:** 1 CITAB, Inov4Agro, University of Trás-os-Montes and Alto Douro (UTAD), Quinta de Prados, 5000-801 Vila Real, Portugal; 2 Department of Forestry Sciences and Landscape Architecture (CIFAP), University of Trás-os-Montes and Alto Douro (UTAD), 5001-801 Vila Real, Portugal; 3 Veterinary and Animal Science Research Centre (CECAV), University of Trás-os-Montes and Alto Douro (UTAD), 5001-801 Vila Real, Portugal; carolina.psca@gmail.com (A.C.A.); mmvpinto@utad.pt (M.V.-P.); 4 Department of Veterinary Sciences, University of Trás-os-Montes and Alto Douro (UTAD), 5001-801 Vila Real, Portugal; raquelrodriguesgoncalvess@gmail.com (R.G.); rui.miranda.mv@gmail.com (R.M.); 5 Idanha-a-Nova Town Hall, 6060-163 Idanha-a-Nova, Portugal; jmqsp14@gmail.com

**Keywords:** geostatistical analysis, GIS, large game, red deer, wild boar, wild ungulates

## Abstract

**Simple Summary:**

Hunting of large game is an activity of great social and economic importance. However, it can cause public health problems due to the zoonotic diseases of these animals, such as tuberculosis. Regular space–time monitoring of hunted animals’ health status allows both the hunters and competent authorities to understand the geographic location of the hunted animals, as well as the occurrence of possible diseases in these animals. This investigation presents the results of the assessment of the spatial–temporal distribution of tuberculosis in large game in a tuberculosis high-risk area in Portugal in the form of maps, which allow any interested party to quickly analyse the hunting situation regardless of their technical or scientific knowledge.

**Abstract:**

Since April 2011, Portugal has implemented specific national legislation (Notice No. 1/2011), defining “Epidemiologic Risk Areas for Bovine Tuberculosis in Large Game” and mitigation measures in these areas, including Idanha-a-Nova county. A GIS project was created to record information that would allow us to analyse the spatial–temporal distribution, both for hunting bags and tuberculosis occurrence, in hunted wild boar and red deer in Idanha-a-Nova. Hunting bag and tuberculosis-like lesion data were recorded during post-mortem inspection across 11 hunting seasons, totalling 9844 animals. The difference in tuberculosis occurrence for these species was statistically significant in nearly all 11 seasons, with wild boars presenting approximately twice the occurrence of red deer. No significant difference was noted before and after the Notice No. 1/2011 implementation. These results, following GIS-based spatial analysis, enable us to state that both large game species displayed an irregular tuberculosis pattern for the 2006–2016 period, and we identified some specific areas of high risk for both species. Southern areas of the county may be considered the priority for intervention. This research demonstrates the potential of GIS tools to evaluate, in the field, the results and efficacy of legislation such as Notice No. 1/2011, and to ensure the correct implementation of cost-effective mitigation strategies for tuberculosis in large game species.

## 1. Introduction

Animal tuberculosis (TB) is a serious animal health issue that has the risk of spreading to humans [1]. TB eradication in cattle is a priority in EU countries [2], and a major concern for the Portuguese Veterinary Authority. Portugal has a robust bovine tuberculosis eradication programme in place. Despite the efforts made over the last few years to eradicate bovine TB, several livestock hotspots remain infected. In some regions, difficulties in eradicating TB in cattle could be related to TB occurrence in wildlife (large game animals), as they share common areas, such as water and feed points, as previously presented by some authors [2,3,4,5]. This epidemiological scenario led the Portuguese National Veterinary Authority to publish, in April 2011, a specific national legislation (Notice No. 1/2011, from the English translation of Editorial No. 1/2011) defining the “Epidemiologic Risk Areas for Bovine Tuberculosis in Large Game”, and describing mandatory rules to be followed in each drive hunt organised within these areas [6].

National scientific research on this subject has been mainly focused on the application of molecular epidemiology techniques, aiming to analyse the possibility of cross-species transmission between wildlife and livestock [7,8,9]. However, to date, no reports have attempted to evaluate the TB spatial–temporal distribution in large game populations in the Portuguese Epidemiologic Risk Areas for Bovine Tuberculosis.

The combined use of Geographical Information System (GIS), Global Navigation Satellite System (GNSS), and Remote Sensing (RS) technologies is being increasingly explored to study the spatial and temporal patterns of infectious diseases, as well as to contribute to decision support models for disease control [3,8,10,11,12,13,14,15].

GIS is an interactive tool with great potential to record, manage, and process geo-referenced information, and to present research results in map format. In wildlife disease research, it is of capital importance to know about animals’ geographical locations, both for endemic disease monitoring and surveillance, and for new disease emergence. GIS integrates spatial analysis tools and geostatistical tools, which allows for analysis of spatial–temporal hunting bag behaviour and monitoring of the effects of disease control strategies [16].

The majority of veterinary research that uses GIS also includes environmental, ecological, topographical, hydrological, climatic, land use, public infrastructure, transportation, and epidemiological data [13,16,17]. In veterinary epidemiology, GIS techniques are crucial and useful, as they offer new analytic opportunities for disease assessment and prevention [18], including assessment of the environmental influence, habitat, and risk factors in disease prevalence [13].

GIS-derived results could be used to predict a disease dynamic via spatial occurrences representation. The geographical patterns could be depicted as a colourful map, based on a disease class interval or a continuous scale derived from descriptive statistics, such as disease prevalence [13,19,20], as in the case of TB in Portuguese large game species. As the geodatabase could be updated regularly, new maps representing the current situation could be regularly and continuously updated, allowing disease spatial dynamic surveillance over time [13]. These are important issues in spatial and temporal sampling schema design, and can support control plan implementation. It is important to decrease the risk of transmission and increase an effective response from the perspective of public health [21].

GIS tools allow the creation of maps and spatial representations that could increase the communication efficacy between authorities and stakeholders with responsibilities in wildlife disease control and effect mitigation, such as risks to public health [19,21].

To the best of the authors’ knowledge, this work is the first geospatial study performed in Portugal to evaluate the spatial–temporal occurrence of TB in large game, supported by Geographic Information Systems (GIS) and geostatistical analyses. Analysing how animals and disease are geographically distributed in the large game population is essential in the comprehension of the TB dynamic in this relevant Portuguese hunting area.

The authors’ main aims for this research were to analyse the spatial–temporal distribution of TB in wild boars and red deer from 2006 to 2016, and the relationship between the hunting bag and the number of TB positive animals—both inter-species and intra-species. The results will be submitted to geostatistical analysis, to identify which areas and species could act as the main source of animal infection, and a major risk for disease transmission, of TB. All results will be compared for the period before and after Notice No. 1/2011 [6] implementation, to assess the biophysical results of this TB mitigation procedure.

A GIS project was created to characterise the study area, to store multi-temporal and spatial information on hunting activities, TB occurrence in wild boar and red deer, and its geographical distribution throughout Idanha-a-Nova county, an important hunting region included in the “Tuberculosis high-risk area in Portugal”. This project also integrated remote sensing capabilities (RS) to use satellite image tasks and global navigation satellite system capabilities (GNSS) to locate post-mortem inspection actions performed in loco by a veterinarian.

## 2. Materials and Methods

### 2.1. Study Area Characteristics

**Figure 1:**
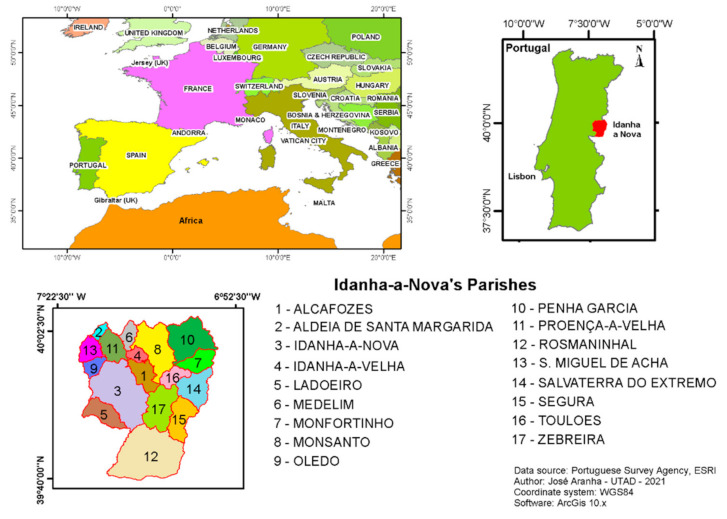
Study area location.

This research project was carried out within Idanha-a-Nova county (Figure 1), which is located in the central-eastern part of Portugal (lat. 39°55′ N; long. 7°14′ W), sharing a border with Spain (Extremadura) and defined by the Erges river on the eastern side and the Tagus river in the south. 

**Figure 2:**
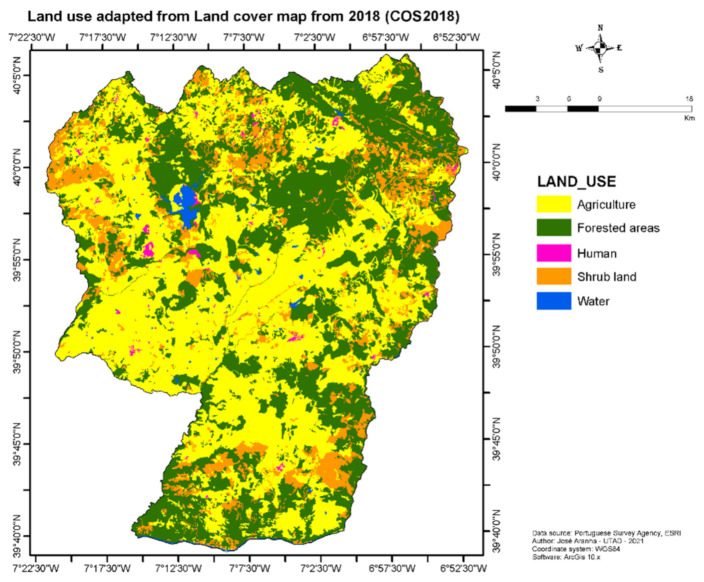
Land-use characteristics of Idanha-a-Nova County in Portugal, adapted from [22].

This is a 1416.25 km^2^ rural area, with low human density. The predominant land covers are agriculture (48.7%), forestry (37.6%), shrubland (12.1%), water (0.9%), and human infrastructure (0.7%) [22], as depicted in Figure 2.

This county is also known as one of the best places for large game hunting in Portugal. In 2020, 121 hunting estates were located within this county, both with fenced game populations (generally with high densities) or free-ranging populations (not fenced), across about 120,000 hectares of hunting area (85% of the county) [22]. This could be associated with the development of an important commercial hunting industry [8].

By force of Notice No. 1/2011, Idanha-a-Nova county is included in the “Portuguese Epidemiologic Risk Areas for Bovine Tuberculosis in Large Game”, due to the high TB prevalence in wild boars and red deer, as already reported in previous research [7,8,15].

### 2.2. Data Collection and Processing

Data were collected from 152 hunting estates across 11 regular hunting seasons (October–February) from 2006 to 2016. Post-mortem inspections of wild boars and red deer was always performed in loco after each drive hunt, by the same veterinarian from the Idanha-a-Nova Town Hall. All hunted animal carcasses and respective viscera were subjected to detailed inspection for gross lesion detection, like the one described by [8,9,23,24,25]. In brief, this involved incision and examination of the retropharyngeal lymph nodes, visual examination and palpation of the lungs, incision and examination of the left bronchial and mediastinal lymph nodes, incision of the mesenteric lymph nodes, and incision of the pre-crural and pre-scapular lymph nodes.

For this study, all caseous or caseoalcareous tubercles with different sizes present in the lymph nodes and organs were considered tuberculosis-like lesions (TBLs). The animal was considered TB positive when at least one TBL was found.

Tuberculosis-like lesion (TBL) occurrence in wild ungulate populations has been employed as a criterion to estimate the occurrence of TB and evaluate the TB distribution [3].

All sampling spots were georeferenced through large game locations using Google Earth and GNSS recorded data.

The population size of individual game species in Idanha-a-Nova was estimated by the reverse calculation method, based on hunting bag statistical analysis [26].

This database was used to calculate the descriptive statistics, Student T-test, Pearson correlation, and for regression analysis [27,28], to analyse the annual hunting bag and TBL occurrence, as well the relationship between these two variables. Non-linear regression models were adjusted by means of the Method of Least Squares (MLS). The adjusted models were than ranked according to the lower Root Mean Square Error (RMSE) and the higher adjusted determination coefficient (R_adj^2^). Statistical analysis and data processing were performed using Microsoft Excel^®^ and the statistical package JMP^®^, from the SAS Institute.

The data regarding the hunting bag and TBL occurrence for wild boars and red deer for each year were submitted for geostatistical analysis using the ArcGIS 10.7 Geostatistical Tool Box, to analyse the spatial hunting bag and TB occurrence spatial distribution within Idanha-a-Nova county [29,30,31,32].

### 2.3. GIS Project

A GIS project was created regarding the wild boar and red deer hunting bag and TBL occurrence each year, from 2006 to 2016, to enable cartographic actions, data processing, geostatistical analysis, and map creation [3,8,11,12,13,15,29,30,31,32].

The database used to derive this research was composed of both binary variables (e.g., sanitary condition: positive or negative to TB) and by quantitative continuous variables (e.g., number of hunted animals, number of positive animals). It was necessary to estimate TB occurrence in large game, both for wild boars and red deer, and to analyse its spatial–temporal distribution, according to the research aims.

Geostatistical analysis was performed for each year in the analyses within Idanha-a-Nova county, and for each species [29,30,31,32]. The calculation was undertaken for the annual hunting bag and the TBL occurrence. The results from the interpolation processes were represented by a colour ramp, which has previously been standardised [30,31,32], to create a continuous map related to the annual hunted animals and disease spread for the Idanha-a-Nova county.

## 3. Results

From 2006 up to 2016, 152 hunting sessions were surveyed in Idanha-a-Nova county, which involved 9844 animals (3963 wild boar and 5881 red deer). Estimation of the TB distribution and occurrence in these ungulate populations was based on TBL detection during post-mortem inspection after each drive hunt. The results are presented in Table 1.

Based on Table 1, it is possible to state that in Idanha-a-Nova county, red deer are more frequently hunted than wild boar but, regarding TBL occurrence, wild boar is the species with the highest values. The results showed that the implementation of Notice No. 1/2011 [6] led to an immediate decrease in TB occurrence in both species.

Student’s T-test was carried out to analyse the statistical significance of TB occurrence within the wild boar hunting bag, within the red deer hunting bag, and between wild boar and red deer, before and after Notice No. 1/2011 [6] was introduced. The results presented in Table 2 show that no statistically significant differences were found for TB occurrence within the same species. Significant results only were found for TB occurrence between wild boar and red deer for 2011 and 2012.

**Figure 3:**
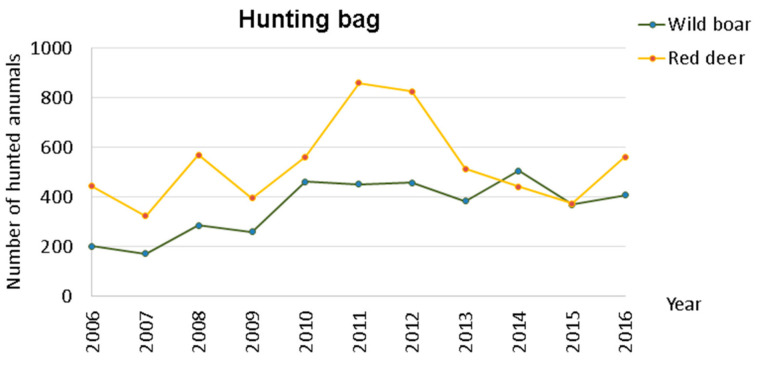
Hunting bag variation within Idanha-a-Nova county from 2006 to 2016.

As previously presented in Table 1, the number of hunted red deer was, in general, higher than the number of hunted wild boar, and this difference was significant at 95% (*t*-student = 2.7897, *p*-value = 0.0126). The graphical representation, Figure 3, shows an alteration in the number of hunted red deer throughout the 11 years, which was opposite to that of wild boar.

**Figure 4:**
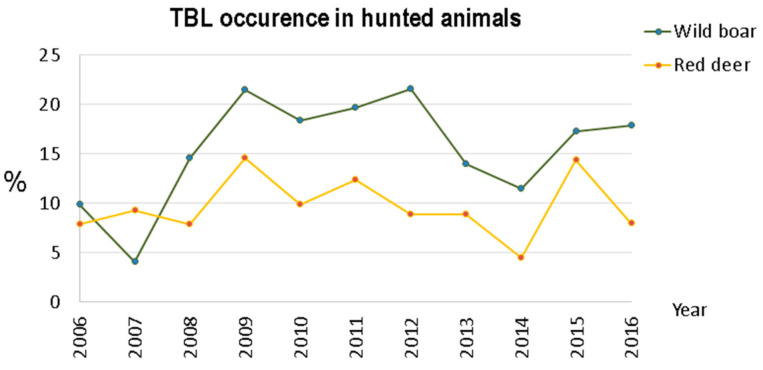
TBL occurrence in the hunting bags within Idanha-a-Nova county from 2006 to 2016.

However, when the global TBL occurrence is calculated for both species (Figure 4), the numbers reveal that occurrence in wild boar is higher than in red deer, and this difference is statistically significant (*t*-student = 3.1095, *p*-value = 0.0067), with wild boars having approximately twice the occurrence.

Both for wild boar and red deer, an irregular disease pattern occurred in the 2006–2016 period. For wild boar, two peaks were observed: the first in 2009, and the second in 2012. The red deer population presented two peaks as well, one in 2009 and a second in 2015. In 2013–2014, we observed a decrease in TBL occurrence in both ungulates.

However, in the years 2015 and 2016, four years after Notice No. 1/2011 [6] was introduced, the TBL occurrence increased again, especially for wild boar. These figures may indicate some relaxation regarding the application of good practices, requiring the implementation of control policies for the effective mitigation of TB in large game species.

In the second stage, the data were subjected to correlation analysis and regression analysis to check whether there was a relationship between the number of hunted animals and the number of TBL-positive animals, both inter-species and intra-species. The results revealed a very significant correlation between the number of hunted animals and the number of TBL-positive animals for both species (*r* = 0.683, *p*-value < 0.05), as presented in Table 3.

These results are in accordance with previously presented results for Spain and France [25,33,34,35], and were adjusted by several regression models [27,28] to the pairs of hunted animals/TBL-positive animals. For both species, the semi-logarithm regression model was the one that better fit these value pairs, as presented in Equations (1) and (2).

Wb_TBL*^+^* = −339.654 + 68.268 × ln(Wb_Ht) (1)

R^2^_adj_ = 0.7492, *p*-value < 0.001

Rd_TBL*^+^* = −298.783 + 56.210 × ln(Rd_Ht) (2)

R^2^_adj_ = 0.4919, *p*-value < 0.01

**Figure 5:**
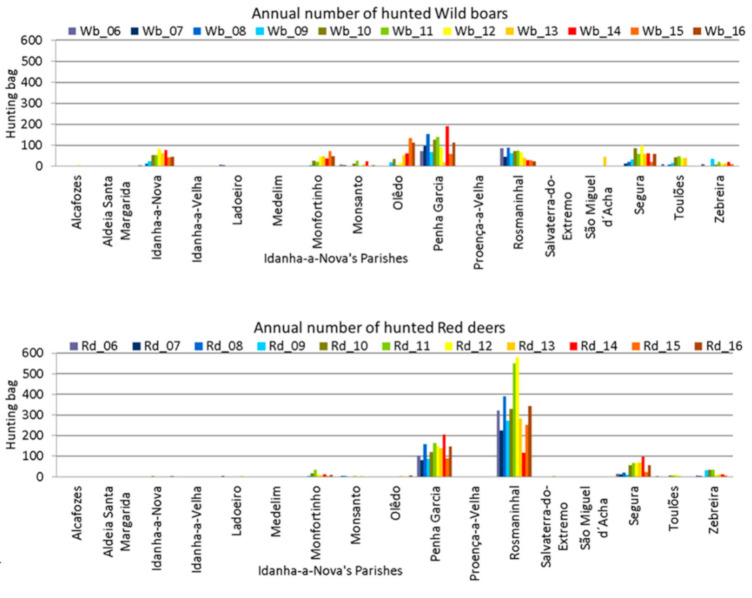
Hunting bag variation across Idanha-a-Nova’s parishes from 2006 to 2016. (**top**): wild boar; (**bottom**): red deer.

Annual totals were disaggregated to the parish level, to enable detection of whether the whole county showed the same behaviour, or if there were discrepancies between parishes. The graphical representations show that wild boars are hunted all over Idanha-a-Nova county (Figure 5, top), but red deer are mainly hunted in three parishes (Figure 5, bottom).

**Figure 6:**
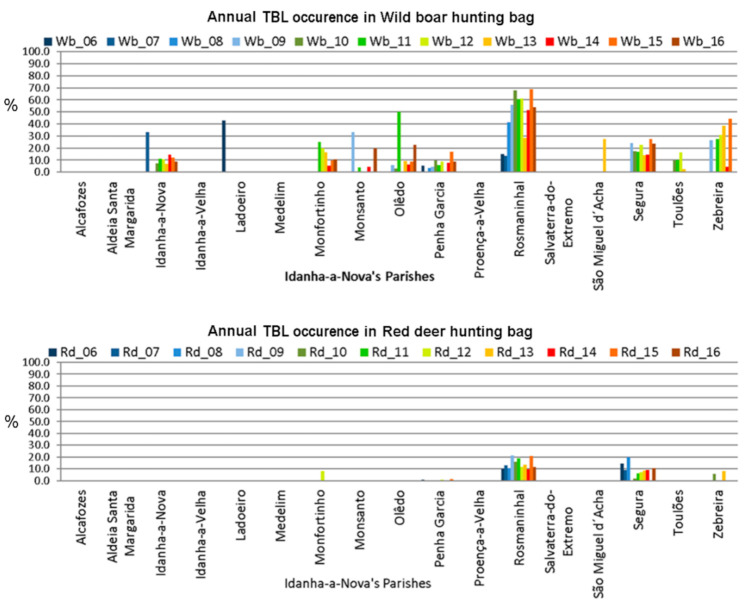
TBL occurrence variation across Idanha-a-Nova’s parishes from 2006 to 2016. (**top**): wild boar; (**bottom**): red deer.

The same disaggregation data were used for TBL occurrence. The graphical representation showed that TBL occurrence in wild boars (Figure 6, top) across Idanha-a-Nova’s parishes seems to be consistent with the hunting bags. On the contrary, TBL occurrence in red deer (Figure 6, bottom) mainly occurs in two parishes: Rosmaninhal and Segura (southern region).

When comparing these two graphical representations by parish, and once we know that in this scenario the contact rate between TB hosts increases equally [28,29], it can be formulated that high densities of animals could increase the potential intra- and inter-species transmission of this infectious agent.

In the third stage analysis, all data were subjected to geostatistical techniques and the Inverse Distance Weighted (IDW) interpolation [29,30,31,32], to create continuous maps related to the hunting bag and TBL occurrence. The IDW method was used as there were only 17 sampling sites available, and this number is not sufficient for the Kriging interpolation process.

**Figure 7:**
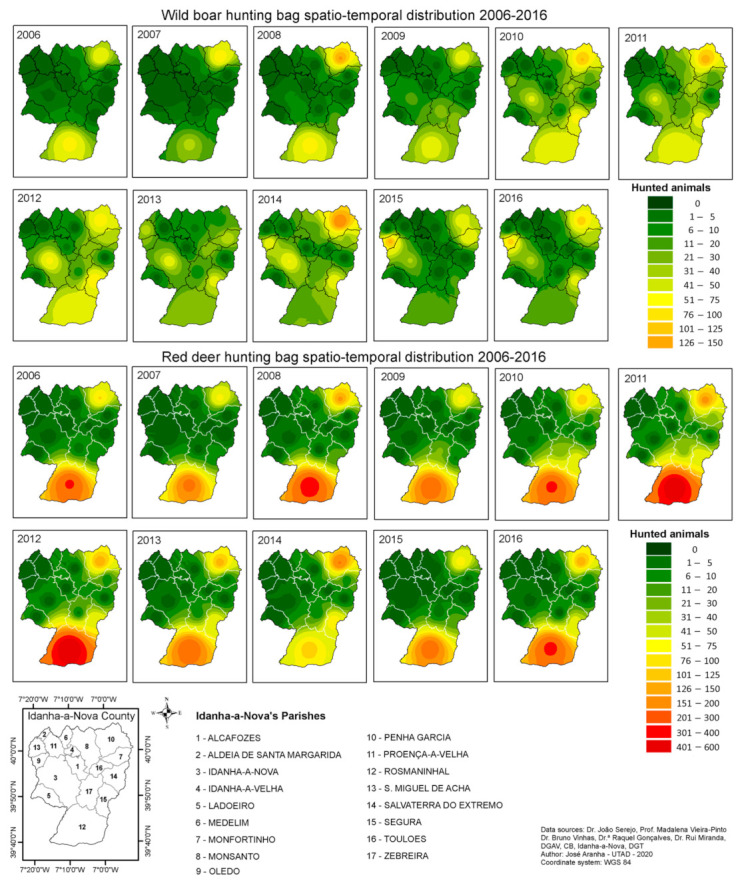
Hunting bag spatial–temporal distribution within Idanha-a-Nova county from 2006 to 2016. (**top**): wild boar; (**bottom**): red deer.

The results, presented in Figure 7 (top: wild boar; bottom: red deer), enable us to state that wild boars are the most widely distributed within Idanha-a-Nova county. The red deer population seems to be more concentrated in the southern and northeastern areas (Rosmaninhal and Segura), and in the northeast (Penha Garcia).

Despite wild boars being present all over the county, it was noted that they also occur in higher numbers in three parishes (Rosmaninhal, Segura, and Penha Garcia).

As shown in Figure 7, the Penha Garcia parish is a hot spot regarding the hunting bag for both species, followed by the Rosmaninhal parish, although it was in this latter parish that more red deer were hunted.

**Figure 8:**
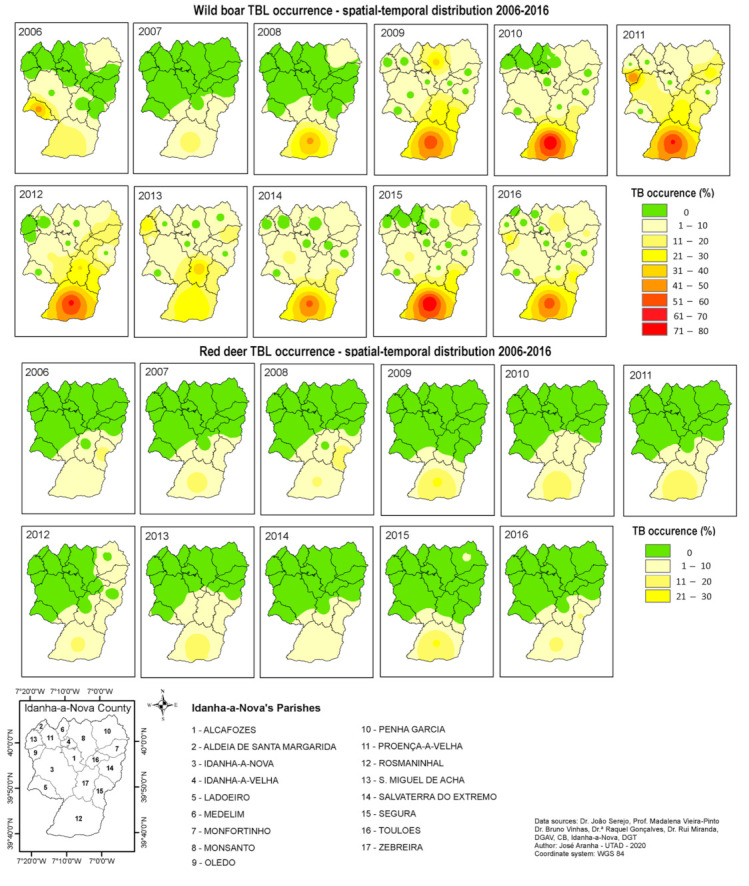
TB occurrence spatial–temporal distribution within Idanha-a-Nova county from 2006 to 2016. (**top**): wild boar; (**bottom**): red deer.

However, it was not in Penha Garcia that more cases of TBL were detected (Figure 8). The most problematic parishes were located in the south: Rosmaninhal and Segura. As aforementioned, this area presented high values of hunting bags for both species, equating to the cohabitation of a large number of animals.

## 4. Discussion

According to previous research [25,33,34,35], high density may favour higher contact rates among TB hosts, which, consequently, may increase TB transmission. Despite our results verifying this statement, we also noted some contradictory outcomes. In the northeast region (Penha Garcia), an equivalently large number of animals presented a lower rate of TBL occurrence in wild boars and just one occurrence in red deer in 2015. Both areas (south and northeast) are hunting states under equivalent game management systems. However, the northeast area is different from the other areas. After GIS-based analysis, it was noted that the northern hunting area was more isolated, with more limited animal movement, due to the presence of a densely forested area and a cliff in the south, which limited animal access from surrounding hunting areas.

This provides additional evidence of a potential interaction between several TB hosts when sharing resources, as suggested in previous research. TB prevalence among red deer was significantly associated with TB prevalence among sympatric wild boar [34], which was confirmed by the correlation value (R = 0.683; *p*-value < 0.05, see Table 2) between TBL-positive wild boar and TBL-positive red deer per annum in this study.

The results demonstrated that the TBL occurrence in wild boars shows an increasing trend in the south–north direction. Wild boar may play an important role in the emergence of new disease foci in distant areas, but red deer do not seem to have the same potential. 

As previously stated by other authors [25,34,35], wild boars seem to be more affected than red deer by a lack of resources, and fences do not impede their movement between estates. Their agility and mobility traits may confer them an important role in TB dispersion within wild or even domestic animals [32]. We noted that the TB occurrence in large game seems to be a severe problem in Idanha-a-Nova county. Presently, it is known that large game species can maintain the disease in the absence of livestock (data not shown), contrary to findings previously described [36,37,38]. The high number of hunted animals in some parishes could be related to some artificial management practices, which could be the cause of the increase in the large game animal population density. This high density may be responsible for the observed increase of TB infection in this population. Infection could occur direct or indirectly, and is observed mainly at risk points for wild animal aggregation [38,39].

In 2011, the Portuguese veterinary authorities implemented good practices in the field, with the internal law Notice No. 1/2011 [6], for the correct management and elimination of by-products related to carcasses from TBL-positive animals. The results here showed that implementation of Notice No. 1/2011 led to an immediate decrease in TBL occurrence in both species, but this decrease was not statistically significant.

However, in two consecutive years (2015 and 2016) after Notice No. 1/2011 [6] introduction, TBL occurrence increased again, mainly within the wild boar population, reflecting the need to evaluate the efficacy of the practices implemented by force of this legislation. The results presented in this technological study may underline the importance of GIS as a tool to support the adoption of effective priority control measures to decrease the TB occurrence in large game in Idanha-a-Nova and in other regions with similar epidemiological conditions.

This research demonstrated the potential of GIS tools to analyse the spatial–temporal distribution of wild animals and TB infection [40], which could support the implementation of cost-effective mitigation strategies [18].

The GIS project could be successively updated, becoming an essential component of the surveillance system in this studied TB hotspot for the Portuguese large game population. This project could elucidate more about the spatial characteristics of this disease by uploading other modelling components: demography, age-structured disease transmission, and a habitat-dependent movement model.

According to some authors [41], predicting the spread of infections among individuals within groups is likely to be relatively straightforward based on patterns of social and mating behaviour, rates of interaction, and sharing of habitats and resources. Other management strategies, such as culling or vaccination, can be also modelled as modifications of these components, and the results demonstrated in representative GIS maps [40,42,43]. These illustrative maps and model outputs could be used to easily communicate with stakeholders and help organise and prioritise management options in coordination with the authorities [43].

## 5. Conclusions

To the best of the authors’ knowledge, this is the first research in Portugal that used information over an extensive period (2006–2016) from GIS techniques to evaluate the spatial–temporal dispersion of TBL in large game. TB in large game is a serious and ongoing problem in Idanha-a-Nova.

Based on the results, we can state that wild ungulates, specifically wild boar, can act as a true reservoir of TB within wildlife in Idanha-a-Nova, and play an important role in disease dispersion. Reducing TB occurrence in large game is urgently necessary, not only due to the risk of cattle transmission, but also due to the negative impact on the hunting industry, as well to protect public health.

Notice No. 1/2011 implementation led to an immediate decrease of TB in both species, but this was not statistically significant. TBL occurrence in the hunting bag presented a similar pattern before and after this attempt to mitigate the problem. An integrated approach should be developed, which must include application of good practices in large game management (suitable densities, fence placement, use of artificial watering, and feeding), classes on the initial examination of hunted game taught by a veterinarian, and adequate elimination of by-products.

As aforementioned, this study identified some specific areas of increased TB risk in Idanha-a-Nova. The southern areas of the county may be considered as the priority for intervention.

To conclude, GIS and geostatistical analysis are essential tools for surveillance systems of infectious diseases in wildlife. This GIS project is constantly being updated, and constitutes an important instrument to continue understanding and monitoring TB transmission and persistence, to help to cost-effectively control and eradicate this multi-host complex zoonotic disease. Thus, further work should be carried out to address a range of important issues, such as data quality, in order to achieve a better understanding of the population biological characteristics, environmental factors, and anthropogenic behaviours related to the disease epidemiology involving this large game population.

## Figures and Tables

**Table 1:** Hunting bags of wild boar and red deer and TB occurrence, for the period 2006–2016.

	Wild Boar			Red Deer		
Year	Hunting Bag (N)	(1) Affected Carcasses (N)	(2) Occurrence (95% C.I.)	Hunting Bag (N)	(1) Affected Carcasses (N)	(2) Occurrence (95% C.I.)
2006	202	20	9.9 (±9.37)	445	35	7.9 (±6.48)
2007	171	7	4.1 (±9.49)	324	30	9.3 (±7.07)
2008	287	42	14.6 (±11.52)	571	45	7.9 (±8.86)
2009	261	56	21.5 (±11.88)	397	58	14.6 (±11.37)
2010	462	85	18.4 (±14.08)	563	56	9.9 (±11.47)
2011	452	89	19.7 (±12.52)	861	107	12.4 (±20.12)
2012	458	99	21.6 (±12.30)	827	74	8.9 (±14.44)
2013	385	54	14.0 (±7.85)	514	46	8.9 (±6.71)
2014	506	58	11.5 (±10.18)	442	20	4.5 (±2.63)
2015	371	64	17.3 (±14.22)	374	54	14.4 (±9.22)
2016	408	73	17.9 (±10.18)	563	45	8.0 (±3.20)
Total	3963	647	-	5881	570	-
Avg.	360.3	58.8	16.4 (±15.14)	534.6	51.8	9.7 (±8.85)
Std.	113.4	28.2		173.5	23.4	

(1) Animals hunted, inspected, and presenting at least one TBL. (2) Occurrence determined by gross pathology. C.I.—Confidence interval; Avg.—Average; Std—Standard deviation. White and grey boxes are related to years before and after application of Notice No. 1/2011, respectively.

**Table 2:** Results of the Student’s *t*-tests for the period 2010–2013.

		Wb_10	Wb_11	Wb_12	Rd_10	Rd_11	Rd_12	Rd_13
Wb_10	*t*-test				1.243			
	*p*-value				0.121			
Wb_11	*t*-test	0.869				2.647		
	*p*-value	0.199				0.011		
Wb_12	*t*-test		0.250				2.151	
	*p*-value		0.403				0.028	
Wb_13	*t*-test			1.101				0.132
	*p*-value			0.142				0.449
Rd_10	*t*-test							
	*p*-value							
Rd_11	*t*-test				0.093			
	*p*-value				0.464			
Rd_12	*t*-test					1.136		
	*p*-value					0.138		
Rd_13	*t*-test						1.343	
	*p*-value						0.099	

Wb—Wild boar; Rd—Red deer.

**Table 3:** Results after correlation analysis for 2006–2016.

Pearson Correlation	Wb_Ht	Wb_ TBL^+^	Rd_Ht	Rd_ TBL^+^
Wild boar hunted (Wb_Ht)	1			
Wild boar TBL positive (Wb_TBL)	0.854 **	1		
Red deer hunted (Rd_Ht)	0.569 ^ns^	0.741 **	1	
Red deer TBL positive (Rd_ TBL)	0.348 ^ns^	0.683 *	0.790 **	1

Statistical significance—ns: non-significant, *p*-value > 0.05; * significant *p*-value < 0.05; ** very significant *p*-value < 0.01.

## Data Availability

Data are reserved information of the author João Serejo, the veterinarian from the Idanha-a-Nova that preformed the post-mortem inspections in loco of wild boar and red deer after each drive hunt.
